# Long-reads-based transcriptome dataset from leaves of lime, *Citrus aurantiifolia* (Christm.) Swingle treated by ethephon and abscisic acid

**DOI:** 10.1016/j.dib.2023.109167

**Published:** 2023-04-18

**Authors:** Elda Kristiani Paisey, Edi Santosa, Ani Kurniawati, Supijatno, Deden Derajat Matra

**Affiliations:** a Agronomy and Horticulture Study Program, Graduate School of IPB University, Bogor, Indonesia; b Agrotechnology Study Program, Faculty of Agriculture, Papua University, Manokwari, Indonesia; c Department of Agronomy and Horticulture, Faculty of Agriculture, IPB University, Bogor, Indonesia

**Keywords:** Abscisic acid, Ethephon, Long-reads sequencing, Self-pruning

## Abstract

The lime plant is a horticultural plant that grows in tropical regions. One of the cultivation maintenances to increase the production of lime fruits is pruning. However, the pruning technique of lime requires high production costs. In addition, phytohormones such as ethylene and abscisic acid have regulation to help drop leaves and branches. The study aimed to identify genes in lime involved in the self-pruning process during ethephon and abscisic acid treatments. Total RNA was extracted and subjected to long-read sequencing using a PCR-cDNA sequencing kit, Oxford Nanopore Technologies. The transcripts were produced 5,914 using the RATTLE program and ranged from 201 – 8,156 bp, and N50 was 1,292 bp. The RNA-seq dataset is available as a raw sequence read that scientists can further process and analyze, and this data can be helpful for lime breeding programs that can shed branches and leaves.

**Specifications Table**

**Table:** 

Subject area	Agronomy and Crop Science
More specific subject area	Fruits
Type of data	RNA sequencing Data
How data was acquired	Data produced using MinION Oxford Nanopore Sequencer
Data format	Raw Sequencing reads, Table, Figure
Description of data collection	RNA sequencing was performed by using MinION Oxford Nanopore. The sample was collected from treated leaves with ethephon and abscisic acid
Data source location	Dramaga, Bogor, West Java, Indonesia
Data accessibility	The data have been archived in the European Nucleotide Archive (ENA) at EMBL-EBI under accession number PRJEB48289 (https://www.ebi.ac.uk/ena/browser/view/PRJEB48289) or https://identifiers.org/insdc.sra:ERP132636 Dataset from leaves of lime (*Citrus aurantiifolia*) by long-read Sequencing, Mendeley Data, V1, doi: https://doi.org/10.17632/52bssfmm4m.1. This dataset consists of two folders, transcriptome assembly and functional annotation.
Related research article	NA

## Value of the Data

• This data provides *Citrus aurantifolia* (Christm.) Swingle transcriptome reference using Oxford Nanopore Technologies of long-read sequencing on ethephon and abscisic acid treatment.
• The presented dataset could help researchers to determine the responses of *Citrus aurantifolia* (Christm.) to ethephon and abscisic acid treatment.
• The data is beneficial for researchers involved in identifying the self-pruning-related genes on *Citrus aurantifolia* (Christm.) under the effects of ethephon and abscisic acid treatment.

## 1 Objective

Lime is a small citrus with a diameter ranging from 4-6 cm and green color with a sour and fresh taste. Lime fruit has anti-inflammatory and anti-bacterial effects [1,2]. Production of lime plants is affected by pruning. Pruning supports vegetative growth, suppresses excessive canopy growth, reduces the impact of alternate bearing, and improves citrus fruit quality. Reduces pest and disease attacks and increases plant cultivation's operational effectiveness [3]. In lime plants, Pruning requires energy, time, and cost, so cultivation techniques are needed that simplify the pruning process. One way is to abort the leaves and branches by themselves based on the induction given in the form of hormones. In addition, self-pruning plays an essential role in flower bud initiation. Previous cytological studies revealed that spring buds in early flowering mutants of trifoliate orange (premature trifoliate orange) initiate differentiation immediately after self-pruning. Several studies have been conducted on genes that play a role in self-pruning, such as in tomatoes [4], sweet oranges (*citrus sinensis*) [5], and grapes [6]. Therefore, it is important to know the effect of growth regulators on the incidence of self-pruning in lime plants. Although, self-pruning can be an alternative to pruning on lime plants. This study aims to make transcriptome references and analyze differences in gene expression that play a role when ABA and ethephon hormones are induced in lime plants. This finding is expected to provide more accurate information about the molecular. The research was conducted through RNA sequencing transcriptome analysis.

## 2 Data Description

In this data, full-length transcripts were sequenced from *Citrus aurantifolia* **(Christm.)** Swingle using long-read sequencing. The total RNA was extracted from the leaves of the seedling stage. The full length as clean-reads was obtained, and de novo assembly was constructed using the RATTLE program. All statistics of reads and assembled transcripts were analyzed (Table 1). The transcripts were annotated with a filtered-UNIPROT database using the BLAST+ v.2.7.1 software. A summary of *Citrus aurantifolia* Swingle Gene Ontology (GO) classification is presented in (Table 2).

**Table 1:** Summary Reads statistics of leaves *Citrus aurantifolia.* Swingle.

	Control	Ethephon	Abscisic Acid
Raw reads	288,110	171,969	159,736
Base raw reads	153,389,860	68,066,407	90,472,258
N50 of raw reads (bp)	612	466	817
Clean reads	275,162	162,408	147,895
Base clean reads	89,399,696	31,528,123	47,924,645
N50 of clean reads (bp)	505	367	735

**Table 2:** Summary of assembly statistics from leaves *Citrus aurantifolia.* Swingle.

Features	Numbers
Clean reads	291,281
Base clean reads (bp)	153,511,017
Total transcripts	5,914
Average (bp)	1,151.02
Largest (bp)	8,156
N50 (bp)	1,292

## 3 Experimental Design, Materials and Methods

**Fig. 1:**
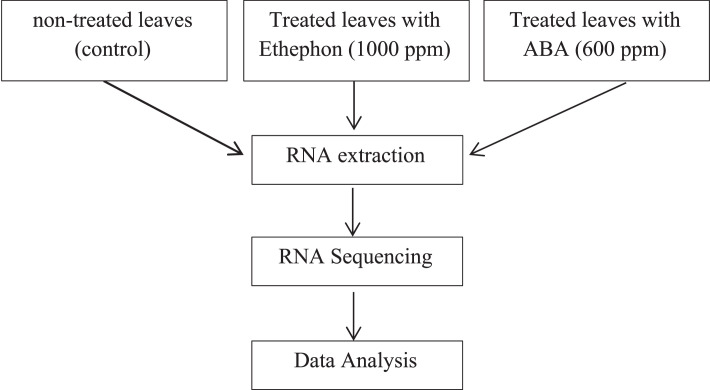
Summary of Experimental Design for RNA Sequencing

*Citrus aurantiifolia (Christm.)* Swingle leaves were collected from a first-year-old lime plant grown in the Leuwikopo Experimental Station, Bogor, West Java, Indonesia (6°33′51.5"S 106°43′29.4"). To conduct RNA sequencing, three types of samples were prepared (Fig. 1). First, the sample was collected from the plant with normal growth as a control, and the lime plant was treated with 1000 ppm ethephon and 600 µM ABA applications. Samples were taken 18 hours after treatment prior to RNA extraction. Next, the total RNA from leaves was extracted using the RNeasy PowerPlant Kit (Qiagen) following the manufacturer's protocol. Next, the quality and quantity of RNA were performed by Qubit™ RNA Broad Range (BR) assay on Qubit® Fluorometer (Invitrogen). Finally, the extracted RNA was conducted for RNA sequencing using PCR-cDNA Barcoding-SQK-PCB109 (PCB_9092_v109_revB_10Oct2019). The sequencing was performed on a Flow Cell R9.4.1 (FLO-MIN106D) on MinION Mk1B. After sequencing, the raw reads were base-called using Guppy 6.1.2 with default parameters. Data pre-processing and de novo assembly were analyzed to obtain full-length transcripts using the RATTLE program [7,8]. The full-length polished transcripts were annotated using the BlastX program [9] with a cut-off of 10^−5^ using the filtered-UNIPROT database (Magnoliopsida (TaxID: 3398), downloaded on 19 October 2021) [10]. Finally, the blasted output was analyzed using Blast2Go software [11,12] (Table 3).

**Table 3:** Functional annotation of transcripts from *Citrus aurantifolia.* Swingle.

Database Source	Number of transcripts (percentage)
UniProt	5173 (87.47%) [12]
GO	4161 (70.36%) [12]
KEGG	107 pathways [12]

## Ethics Statements

No animal or human experiments were performed in this study.

## CRediT authorship contribution statement

**Elda Kristiani Paisey:** Conceptualization, Methodology, Writing – original draft, Funding acquisition. **Edi Santosa:** Conceptualization, Validation, Investigation, Writing – review & editing, Funding acquisition. **Ani Kurniawati:** Supervision, Writing – review & editing. **:** Supervision, Writing – review & editing. **Deden Derajat Matra:** Supervision, Writing – review & editing, Funding acquisition.

## Declaration of Competing Interest

The authors declare that they have no known competing financial interests or personal relationships that could have appeared to influence the work reported in this paper.

## Data Availability

Dataset from leaves of lime (Citrus aurantiifolia) by long-read Sequencing (Original data) (Mendeley Data).
